# A novel fatty-acid metabolism-based classification for triple negative breast cancer

**DOI:** 10.18632/aging.204552

**Published:** 2023-02-25

**Authors:** Xia Yang, Wen Tang, Yongtao He, Huimin An, Jin Wang

**Affiliations:** 1 Department of Pathology, Sir Run Run Shaw Hospital of Zhejiang University School of Medicine, Hangzhou, China

**Keywords:** fatty acid metabolism, tumor microenvironment, immunotherapy, triple negative breast cancer

## Abstract

Background: The high heterogeneity of triple negative breast cancer (TNBC) is the main clinical challenge for individualized therapy. Considering that fatty acid metabolism (FAM) plays an indispensable role in tumorigenesis and development of TNBC, we proposed a novel FAM-based classification to characterize the tumor microenvironment immune profiles and heterogeneous for TNBC.

Methods: Weighted gene correlation network analysis (WGCNA) was performed to identify FAM-related genes from 221 TNBC samples in Molecular Taxonomy of Breast Cancer International Consortium (METABRIC) dataset. Then, non-negative matrix factorization (NMF) clustering analysis was applied to determine FAM clusters based on the prognostic FAM-related genes, which chosen from the univariate/multivariate Cox regression model and the least absolute shrinkage and selection operator (LASSO) regression algorithm. Then, a FAM scoring scheme was constructed to further quantify FAM features of individual TNBC patient based on the prognostic differentially expressed genes (DEGs) between different FAM clusters. Systematically analyses were performed to evaluate the correlation between the FAM scoring system (FS) with survival outcomes, genomic characteristics, tumor microenvironment (TME) features and immunotherapeutic response for TNBC, which were further validated in the Cancer Genome Atlas (TCGA) and GSE58812 datasets. Moreover, the expression level and clinical significancy of the selected FS gene signatures were further validated in our cohort.

Results: 1860 FAM-genes were screened out using WGCNA. Three distinct FAM clusters were determined by NMF clustering analysis, which allowed to distinguish different groups of patients with distinct clinical outcomes and tumor microenvironment (TME) features. Then, prognostic gene signatures based on the DEGs between different FAM clusters were identified using univariate Cox regression analysis and Lasso regression algorithm. A FAM scoring scheme was constructed, which could divide TNBC patients into high and low-FS subgroups. Low FS subgroup, characterized by better prognosis and abundance with effective immune infiltration. While patients with higher FS were featured with poorer survival and lack of effective immune infiltration. In addition, two independent immunotherapy cohorts (Imvigor210 and GSE78220) confirmed that patients with lower FS demonstrated significant therapeutic advantages from anti-PD-1/PD-L1 immunotherapy and durable clinical benefits. Further analyses in our cohort found that the differential expression of CXCL13, FBP1 and PLCL2 were significantly associated with clinical outcomes of TNBC samples.

Conclusions: This study revealed FAM plays an indispensable role in formation of TNBC heterogeneity and TME diversity. The novel FAM-based classification could provide a promising prognostic predictor and guide more effective immunotherapy strategies for TNBC.

## INTRODUCTION

Triple-negative breast cancer (TNBC), as defined by the absence of estrogen receptor (ER) and progesterone receptor (PR), and human epidermal growth factor receptor 2 (HER2), is a heterogeneous breast cancer subtype that carries the worst prognosis due to its aggressive characteristics and limited therapeutic options [1, 2]. Much effort has been devoted over the past decade in classifying TNBCs into several molecular subtypes with distinct mutational profiles, genomic alterations, and biological processes that could guide treatment decisions [3–7].

Lipid metabolism, especially the synthesis of fatty acids (FAs), is an important cellular process that converts nutrients into metabolic intermediates for membrane biosynthesis, energy storage, and signal molecule production [8]. Abnormal lipid metabolism is one of the hallmarks of cancer. Increasing evidence confirmed the significance role of FAM in carcinogenesis, affecting cell–matrix interaction, cell signaling and communication, as well as tumor angiogenesis and metastasis, and immune modulation [9, 10]. Targeting FAM process has become a promising therapeutic strategy for tumors [11, 12]. However, researches of FAM-relevant molecular stratification in TNBC have not yet been established.

Recently, immune checkpoint inhibitors (ICIs) has achieved an impressive clinical response in various types of cancer, whereas the overall response rate and clinical benefit rate is still unsatisfied in TNBC [13–15]. The biological and functional heterogeneity of T cells is a pivotal determinant for effective antitumor immunity and immunotherapy. Therefore, it is critical to further elucidate the molecular mechanism of T cell dysfunction in tumor microenvironment.

The mechanism of Immunometabolism in regulating the function and fate of immune cells has been widely concerned. Previous studies found that tumor cells and Treg cells drove elevated expression of group IVA phospholipase A2, then altered lipid metabolism and senescence of T cells. Inhibition of group IVA phospholipase A2 reprogrammed effector T cell lipid metabolism, effectively prevented T cell senescence, and enhanced anti-tumor immunity and immunotherapy efficacy [16]. Meanwhile, S-palmitoylation, a lipid process that covalently binds palmitic acid to protein residues, has been found to play an indispensable role in maintaining PD-L1 stability and inhibiting T cell cytotoxicity [17]. However, the specific effects of FAM on the tumor microenvironment immune profiles in TNBC are not fully studied.

In this study, we comprehensively evaluated the association between FAM and TME cell-infiltrating characteristics and heterogeneous by integrating the transcriptomic and genomic data of 470 TNBC samples from METABRIC, TCGA and GEO databases. Firstly, FAM-related genes were identified by applying WGCNA in TNBC patients. Then, 3 distinct FAM clusters with nonnegative matrix factorization (NMF) clustering were identified. Moreover, we constructed a scoring scheme to quantify the FAM features in individual TNBC patient. The prognosis traits, genomic variations, transcriptome features, as well as immune infiltration among the different FAM subtypes were further analyzed and verified. These findings indicated that FAM plays a crucial role in reshaping the heterogeneous and tumor immune microenvironment in TNBC.

## METHODS

### Data acquisition and preparation

The workflow was shown in Supplementary Figure 1. TNBC patients with full clinical annotations and RNA-seq data were searched from Molecular Taxonomy of Breast Cancer International Consortium (METABRIC, http://www.cbioportal.org/datasets), the Cancer Genome Atlas (TCGA, https://portal.gdc.cancer.gov/repository), and Gene-Expression Omnibus (GEO, http://www.ncbi.nlm.nih.gov/geo/) datasets. In total, 470 TNBC samples were included in our study for further analysis. The microarray data of the METABRIC (*N* = 221) was served as training dataset. The normalized RNA-seq data which acquired from TCGA database (*N* = 142) and the gene expression profiles from GSE58812 (*N* = 107) were used as independent validation datasets. R package ‘limma’ was applied for gene expression normalization [18].

### Identification of FAM-related genes

The hallmark gene sets of fatty acid metabolism (FAM), which including 158 FAM relevant genes, were extracted from the Molecular Signatures Database (MSigDB) (https://www.gseamsigdb.org/gsea/msigdb/). First, we calculated the FAM ssGSEA score in METABRIC-TNBC samples by the ssGSEA algorithm (R package “gsva”) [19]. Then, we screened out the FAM module and FAM genes by the R package “wgcna” [20]. Pearson’s correlation matrices, co-expression similarity matrix, and average linkage method were performed to estimate the correlation coefficient between any two genes. A weighted adjacency matrix with a scale free co-expression network and topological overlap matrix (TOM) were constructed to investigate the connectivity and dissimilarity of the co-expression network. A hierarchical clustering tree was established based on the TOM dissimilarity, which could identify the key modules and genes. Then, we set the module membership (MM) >0.8 and gene significance (GS) > 0.5 to identify the correlation between genes and FAM ssGSEA score. Totally, 1860 candidate FAM-related genes were identified from the FAM module.

### Prognostic value analysis

The prognostic significance of the FAM-related genes which obtained from the WGCNA was analyzed by univariate Cox regression model using the R package “survival”. Then, LASSO Cox regression algorithm was applied to further select prognostic FAM genes using the R package “glmet” [21]. Subsequently, the most robust prognostic gene signatures for OS were chosen by performing multivariate Cox regression model in METABRIC-TNBC patients.

### Non-negative matrix factorization (NMF) clustering analysis

Based on the expression of 8 prognostic FAM-related genes, we then applied NMF clustering analyses to identify distinct FAM clusters. The optimal number of clusters and their stability were determined by the consensus clustering algorithm. The R package “NMF” was used to perform the consensus clustering [22].

### Identification of differentially expressed genes (DEGs) between distinct FAM clusters

DEGs between distinctFAM clusters were identify using the R package “limma” [18]. The significance criteria for determining DEGs was set as adjusted *P* value < 0.01.

### Gene set variation analysis (GSVA) and functional annotation

GSVA enrichment analysis using “GSVA” R packages were performed to investigate the variation in biological process between different FAM clusters [19]. The gene sets of “c2.cp.kegg.v7.1.symbols” were downloaded from MSigDB database for running GSVA analysis. Adjusted *P* value < 0.05 was considered as statistically significance. The “clusterProfiler” R package was used to perform functional annotation for DEGs between different FAM clusters, with the cutoff value of FDR < 0.05 and *P* < 0.05 [23].

### Estimation of TME cell infiltration

ssGSEA algorithm was used to quantify the relative abundance of 28 immune cell types in the TME [24, 25]. CIBERSORT algorithm was applied to analyze the components of 22 immune cell types among different FAM subgroups [26]. MCP-counter was performed to show the immune-related activity among different FAM subgroups using the ‘MCPcounter’ package [27]. The immune and stromal compositions in TME were further estimated using the ‘estimate’ package [28]. Moreover, the expression of key immune profiles was compared between different FAM clusters.

### Generation of a novel FAM-based classification

To quantify the FAM features of individual TNBC patient, we explored a novel FAM-based classification—the FAM scoring system (FS) to investigate the FAM features of individual TNBC patient. Specifically, 782 overlap DEGs were identified from different FAM clusters, we then extracted the prognostic gene signatures using univariate Cox regression model and Lasso regression algorithm. Finally, 8 genes were chosen to construct the FAM scoring system. The FAM Score (FS) was calculated by the corresponding coefficients of selected gene signatures:

FAM Score =Σ*i* Coefficient (mRNA) × Expression (mRNA)

Where *i* represent the selected gene signatures.

### Gene set variation analysis (GSVA)

We then performed GSVA to further reveal the most significantly enriched molecular pathways between different FS subgroups using the R package “GSVA” [19]. The gene sets of “c2.cp.kegg.v7.1.symbols” were downloaded from MSigDB database. Adjusted *P* value < 0.05 was considered as statistically significance.

### Significantly mutated genes and tumor mutation burden in different FS groups

Principal component analysis (PCA) for the expression profiles of 8 FS gene signatures were analyzed and presented between tumor and normal samples in TCGA cohort. Moreover, the CNV variation frequency of 8 FS gene signatures were further evaluated in TCGA-TNBC cohort. The R package of RCircos was applied to depict the copy number variation landscape of these selected gene signatures in 23 pairs of chromosomes [29]. Using the R package maftools [30], the overall mutation landscape was summarized and present in patients with high and low FS subgroups in TCGA cohort. Based on the TGCA somatic mutation data, we then calculated TMB scores to assess the mutation status between high and low FS subgroups.

### Genomic and clinical data sets with immune-checkpoint blockade

The immunotherapeutic cohorts: IMvigor210 cohort (advanced urothelial cancer treated with atezolizumab, anti PD-L1 antibody) [31] and GSE78220 cohort (metastatic melanoma with intervention of pembrolizumab, an anti PD-1 antibody) [32] were chosen to analyze the predictive efficiency of FS scheme for immunotherapy.

### Exploration of potential compounds targeting the selected FAM-related gene signatures

To explore potential compounds targeting the selected FS-related gene signatures for treatment of TNBC, we calculated the therapeutic response of various molecular based on their half-maximal inhibitory concentration (IC50) which extracted from the CellMiner database [33].

### Validation of the bioinformatics results in clinical samples

We first collected 63 paired TNBC tissues and adjacent normal tissues (ANT) from Sir Run Run Shaw hospital of Zhejiang University School of Medicine. Total RNAs was extracted with TRIzol reagent (Invitrogen, USA) according to the manufacturer’s instruction, which then reverse transcribed into complementary DNA using PrimeScript RT MasterMix (Takara, China). Using SYBR Green PCR MasterMix (Takara, China), RT qPCR was performed according to the manufacturer’s instruction. The primers were listed in (Supplementary Table 1). The average Ct value were used for each gene which repeated three times, β-actin was applied to normalize the target genes mRNA expression.

### Statistical analysis

Student’s *t*-tests were applied to analyze normally distributed variables, Wilcoxon rank-sum tests were used to estimate non-normally distributed variables. One-way ANOVA and Kruskal-Wallis tests were performed to examine difference comparisons of more than two groups. Kaplan-Meier curve and Cox proportional hazards model were chosen to investigate the prognostic significance of FAM-related genes and FAM subtypes. All statistical analyses were done in R 4.0.1 software. Statistical significance was determined with a two-sided *p* < 0.05.

### Availability of data and materials

All data used in this work can be acquired from the Gene-Expression Omnibus (GEO; https://www.ncbi.nlm.nih.gov/geo/) under the accession number GSE78220 and GSE58812, METABRIC and the TCGA portal (https://portal.gdc.cancer.gov/).

## RESULTS

### Identification of FAM-related module and genes

**Figure 1:**
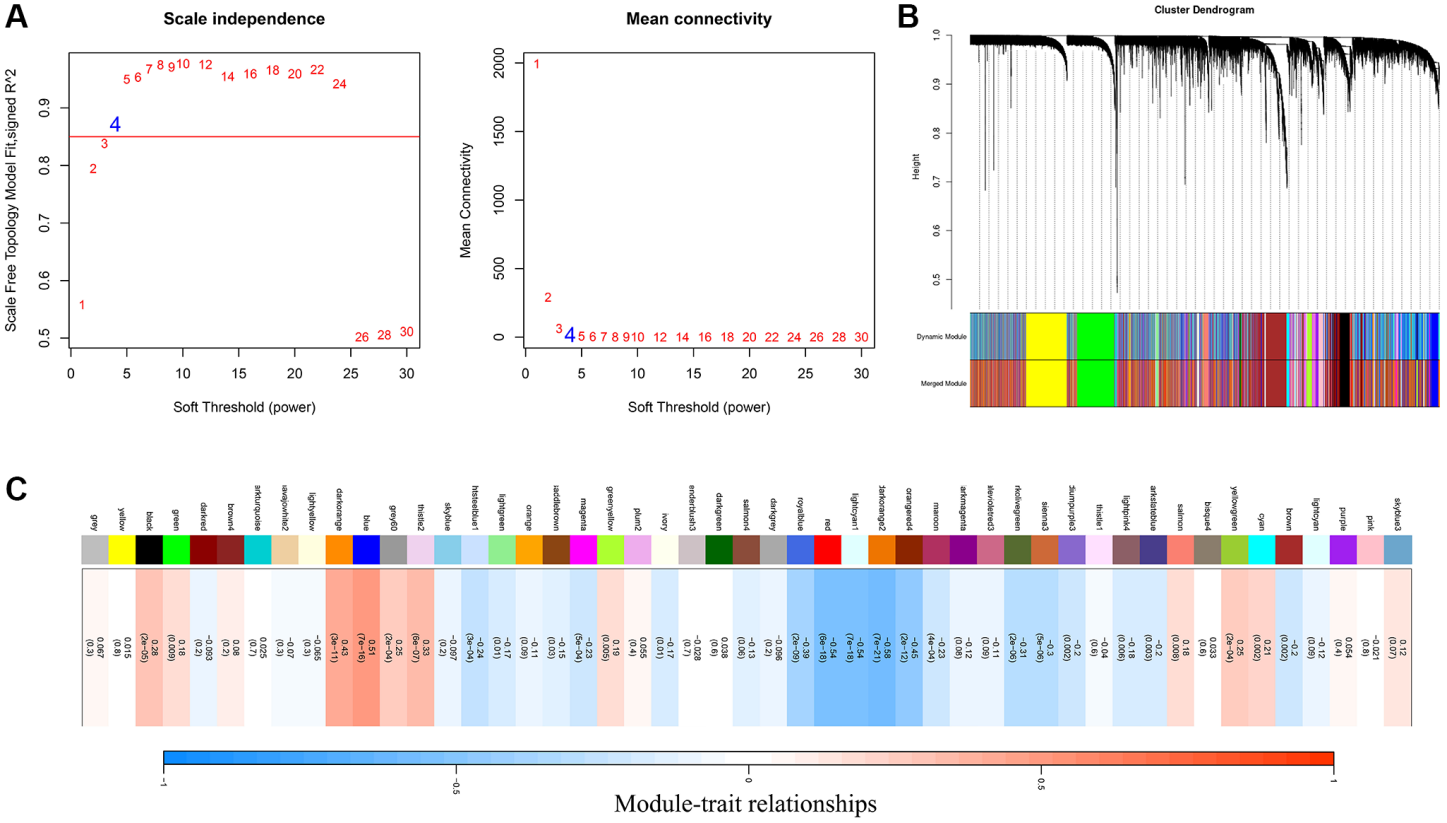
**Identification of FAM-related module and genes.** (**A**) The optimal soft threshold power was chosen as β = 4. (**B**) A hierarchical clustering tree was established to identify the FAM-related module. (**C**) The blue module was identified positively correlated with FAM, which we termed as FAM module.

Based on the FAM ssGSEA score we calculated by the ssGSEA algorithm, a gene co-expression network was constructed with the WGCNA algorithm to identify FAM-related module. The most critical parameter of the soft threshold power was set at 4 (Figure 1A). Then, a hierarchical clustering tree was established to identify the key modules and genes (Figure 1B). Figure 1C showed that the blue module was positively correlated with FAM, which termed as FAM-related module, and the genes in the blue module were regarded as FAM-related genes (*n* = 1860).

### Different FAM clusters mediated by FAM-related genes

**Figure 2:**
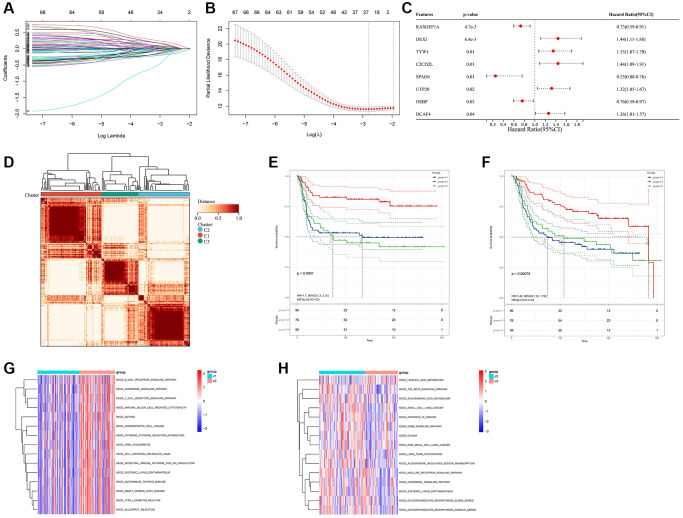
**Different FAM clusters mediated by FAM-related genes.** (**A**) 78 prognostic FAM genes were further selected by Lasso regression algorithm. (**B**) 8 prognostic FAM-related genes were finally identified applying multivariate Cox regression model. (**C**) Forest plot visualize the prognostic value of 8 prognostic FAM-related genes. (**D**) Three distinct FAM clusters were established based on the expression of prognostic FAM genes using Consensus Clustering analysis of the NMF algorithm. Survival analyses for RFS (**E**) and OS (**F**) among different FAM clusters in METABRIC-TNBC cohort. (**G**) Differences in KEGG pathways between the FAM cluster-C1 and FAM cluster-C3. (**H**) Differences in KEGG pathways between the FAM cluster-C2 and FAM cluster-C3.

Prognostic analysis (including univariate/multivariate Cox regression model and Lasso regression algorithm) were performed to identify the prognostic values of these FAM-related genes (Figure 2A–2C and Supplementary Table 2). Then, Consensus Clustering analysis of the NMF algorithm were applied to classify patients with qualitatively different FAM clusters based on the expression of prognostic FAM-related genes (Figure 2D). Three distinct FAM clusters were eventually identified, including 90 cases in FAM cluster C1, 76 cases in FAM cluster C2 and 55 cases in FAM cluster C3 (Supplementary Table 3). Prognostic analysis revealed that particularly prominent survival advantage in FAM cluster-C2, whereas the worst prognosis found in FAM cluster-C3 (Figure 2E, 2F). GSVA algorithm showed significant differences in KEGG pathways among these distinct FAM clusters (Figure 2G, 2H).

### TME cell infiltration characteristics in distinct FAM clusters

**Figure 3:**
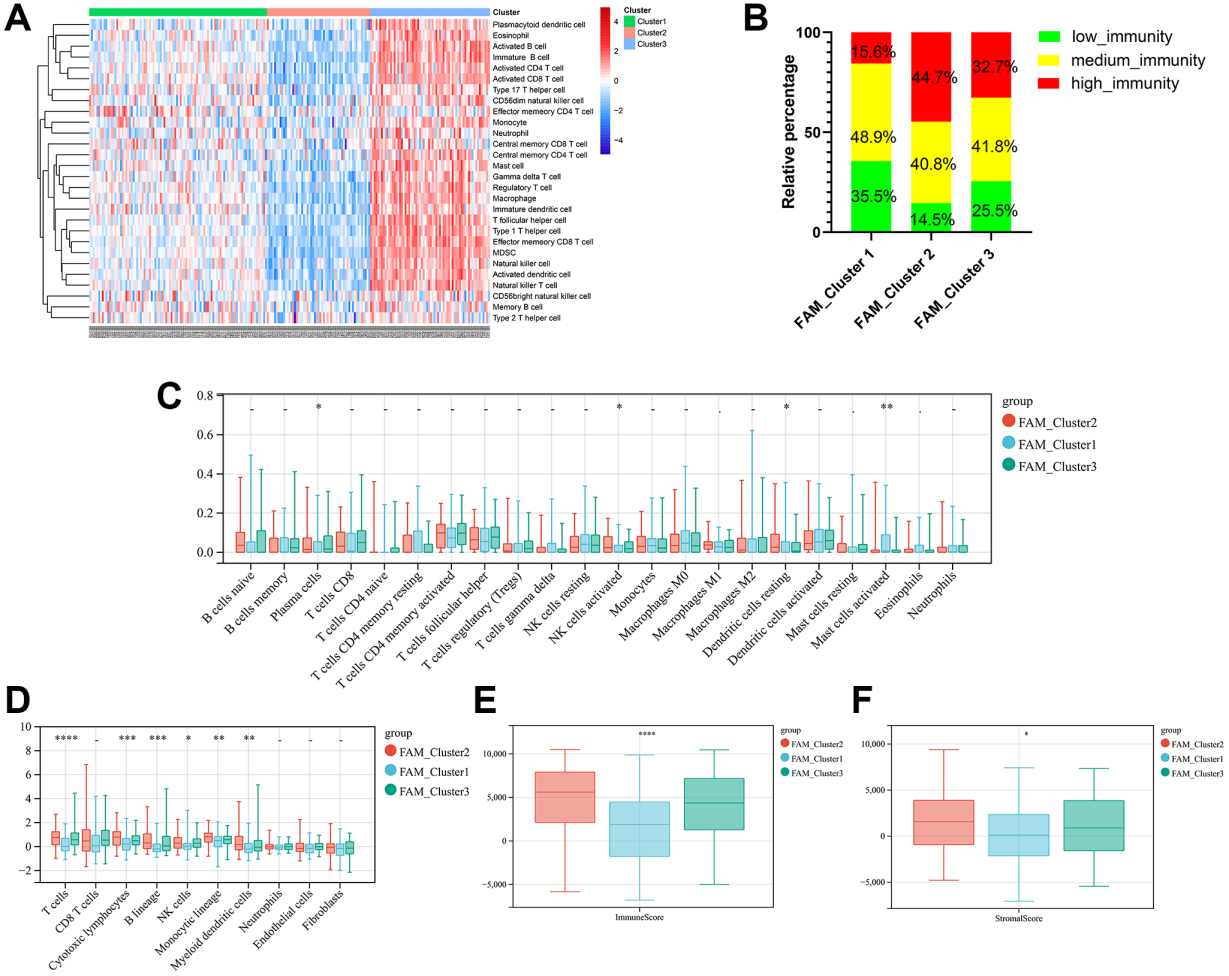
**TME cell infiltration characteristics in distinct FAM clusters.** (**A**) ssGSEA showed three distinct immunity phenotypes were identified in METABRIC-TNBC cohort. (**B**) The rate of different immunity phenotypes among different FAM clusters. (**C**) Cibersort revealed the abundance of each TME infiltrating cells among different FAM clusters. (**D**) MCPcounter revealed the abundance of each immune infiltrating cell types among different FAM clusters. (**E**, **F**) ESTIMATE analysis exhibited the diversity of the immune (**E**) and stromal score (**F**) among different FAM clusters.

Firstly, we analyzed the enrichment levels of 28 immune signatures in the TME of TNBC by performing ssGSEA, METABRIC-TNBC samples were further classified into three distinct immune subtypes (Figure 3A). 67 patients were set at the high-immunity group, which characterized by abundance of immune cell infiltration; The low-immunity group contained 57 patients, which characterized by the suppression of immunity, and 97 patients were present in the modulate-immunity group, which represented with inadequate immune cell infiltration and ineffective antitumor immunity. Then, we analyzed the distribution of immune signatures among these distinct FAM clusters. As shown in Figure 3B, FAM cluster-C2 was markedly associated with high-immunity group, whereas FAM cluster-C1 presented high proportion of low-immunity group. Then, CIBERSORT algorithm and MCPcounter method were used to show the differences on the component of TME immune cell profiles between distinct FAM clusters. As shown in Figure 3C, 3D, FAM cluster-C2 was remarkably enriched in innate immune cell infiltration including natural killer cell, plasma cells, Myeloid dendritic cell, and cytotoxic lymphocytes compared to other FAM clusters. Moreover, ESTIMATE analysis found that the diversity distribution of the immune and stromal scores between different FAM clusters, which suggested that FAM plays an inevitable role in tumor microenvironment immune profiles (Figure 3E, 3F).

### Prognostic DEGs between different FAM clusters

**Figure 4:**
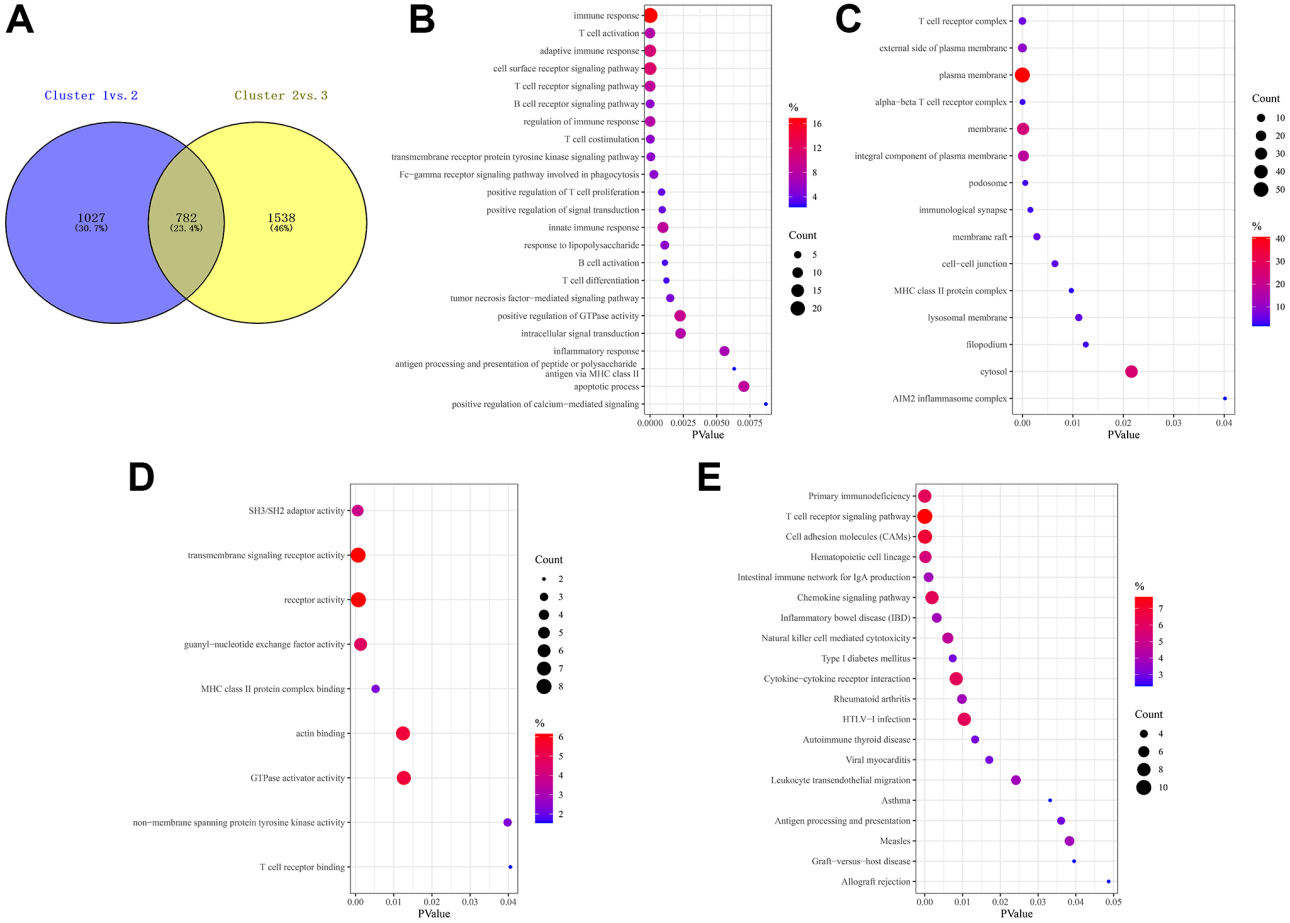
**Prognostic DEGs between different FAM clusters.** (**A**) 782 DEGs were identified across three FAM clusters using the R package “limma”. (**B**–**E**) Functional annotation for these DEGs using GO enrichment analysis, with the enrichment of GO-BP (**B**), GO-CC (**C**), GO-MF (**D**) and KEGG pathways of the 140 prognostic DEGs using univariate Cox regression analysis.

Considering the prominently prognostic difference among the FAM-clusters, we further examined the potential FAM-related transcriptional expression change across three FAM clusters in METABRIC-TNBC samples. A total of 782 DEGs were identified, which depicted in Figure 4A and Supplementary Table 3. Univariate Cox regression model based on the 782 DEGs was performed to find prognostic FAM-related DEGs (Supplementary Table 3). Functional enrichment analysis of these prognostic FAM-related DEGs revealed that 49 biological processes (BP) related to immune response and T cell activation, 23 cellular components (CC), including T cell complex and plasma membrane, and 14 molecular functions (MF) refer to SH3/SH2 adaptor activity and transmembrane signaling receptor activity were significant enriched; 13 KEGG pathways related to primary immunodeficiency and T cell receptor signaling pathway were significant over-represented (Figure 4B–4E).

### Construction of FAM gene signature and FAM scoring system

**Figure 5:**
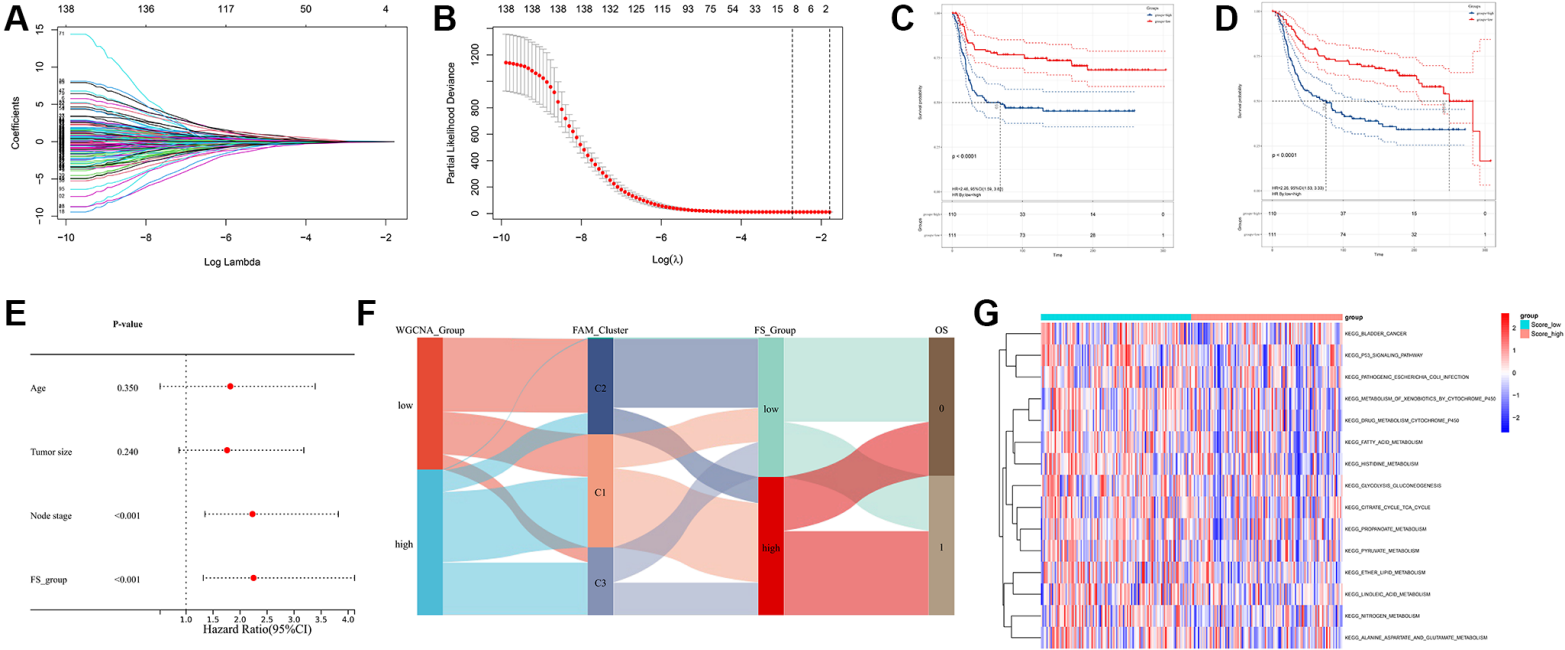
**Construction of FAM gene signature and FAM scoring system.** (**A**, **B**) Lasso Cox regression algorithm found the prognostic genes from the 140 prognostic DEGs. (**C**, **D**) Survival analyses for RFS (**C**) and OS (**D**) between low- and high- FS groups in METABRIC-TNBC cohort. (**E**) Multivariate Cox regression analysis confirmed that FS could serve as an independent prognostic biomarker for METABRIC-TNBC samples. (**F**) Alluvial diagram showing the changes of FAM clusters, FS subtypes and overall survival. (**G**) Differences in KEGG pathways between the low- and high FS groups.

Based on the obtained 140 prognostic FAM-related DEGs, Lasso regression analysis was performed to stratify TNBCs into different genomic subtypes (Figure 5A, 5B and Supplementary Table 3). Totally, 8 selected FAM-related prognostic genes were identified, which were defined as FAM-related gene signatures (Supplementary Table 3). Then, a set of FAM scoring system, which termed as FAM score (FS), was established to quantify the FAM features of individual TNBC patient. Consistent with the clustering grouping of FAM clusters, two distinct FAM score subgroups were found and we named these 2 subgroups as FS-low and -high. Kaplan-Meier survival analysis shown that significant prognostic differences between the low- and high- FS subgroups (Figure 5C, 5D), patients with lower FS was shown with better survival outcomes. Furthermore, multivariate Cox regression model analysis confirmed that the FS scheme could serve as an independent prognostic biomarker for METABRIC-TNBC patients (Figure 5E and Supplementary Table 4). To better illustrate the association between FAM features with prognosis, we visualized the attribute variety of individual TNBCs by applying alluvial diagram (Figure 5F). Moreover, significant differences were observed between the low and high FS groups refer to KEGG pathways (Figure 5G).

### TME immune cell profiles between distinct FS subgroups

**Figure 6:**
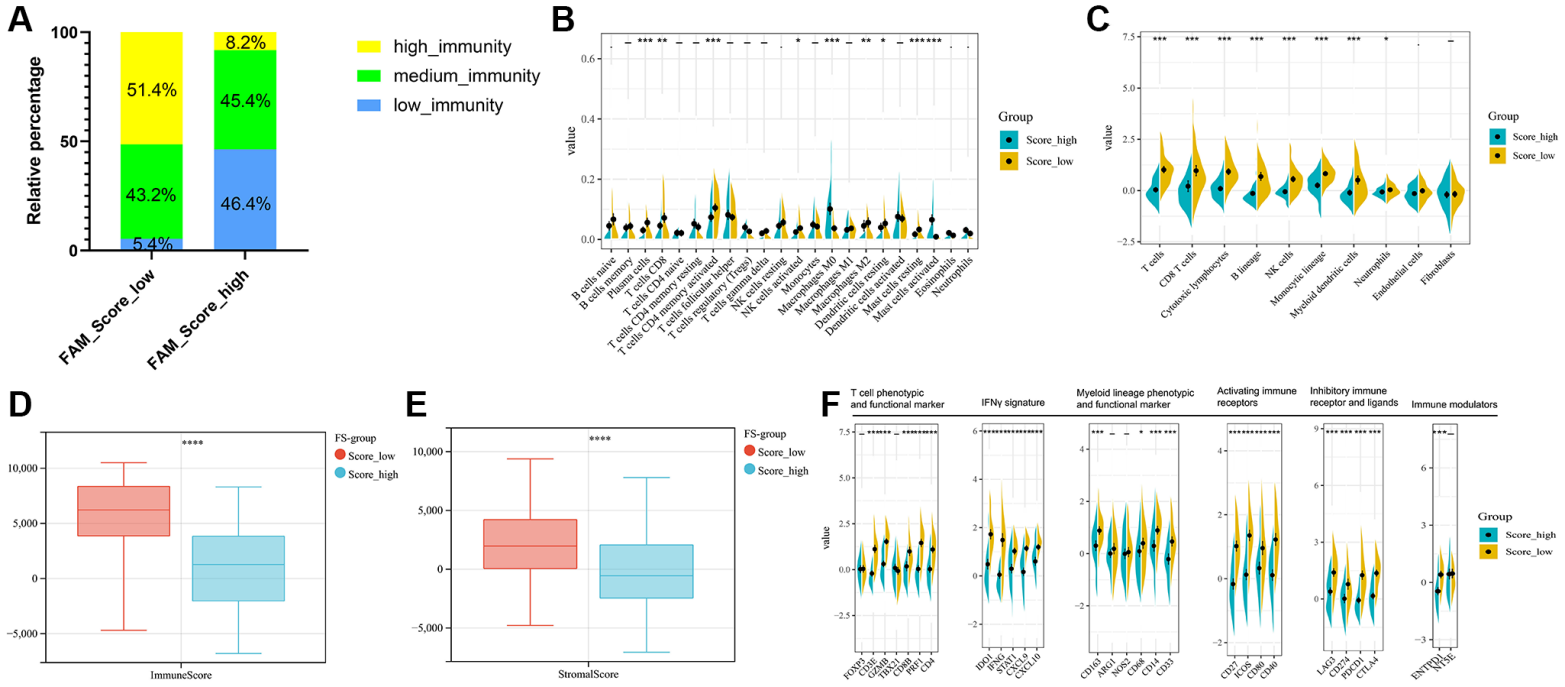
**TME cell infiltration characteristics in distinct FS subgroups in METABRIC-TNBC cohort.** (**A**) The rate of different immunity phenotypes between the low- and high FS groups. (**B**) Cibersort revealed the abundance of each TME infiltrating cells between the low- and high-FS groups. (**C**) MCPcounter revealed the abundance of each immune infiltrating cell types between the low- and high-FS groups. (**D**, **E**) ESTIMATE analysis exhibited the diversity of the immune (**D**) and stromal score (**E**) between the low- and high-FS groups. (**F**) The expression of immune profiles between the low- and high-FS groups in METABRIC-TNBC cohort.

Regarding the immune classification, low-FS subtype consisted of more proportions of high- and medium-immunity tumors, whereas high-FS subtype contained mainly low-immunity tumors. (Figure 6A). The results of Cibersort and MCPcounter methods revealed the different infiltrating abundances of TME immune infiltrating cell types between the low- and high-FS groups. As shown in Figure 6B, 6C, low-FS subtype was remarkably abundant with activate immune cell infiltration including cytotoxic lymphocytes, plasma cells, CD8+ T cells, NK cells, Myeloid dendritic cell, and mast cells compared to the high FS subtype. ESTIMATE analysis exhibited the diversity of the immune and stromal scores between distinct FS subgroups (Figure 6D, 6E). Moreover, significant reverse correlation between the abundance of immune profiles and FS were found in METABRIC-TNBC cohort (Figure 6F). The above findings demonstrated that low-FS subtype tumors had relatively higher immune infiltration levels compared with that in high-FS subtype.

### Clinical application of the FAM scoring system in two independent cohorts

To further explore the clinical application value of the novel FAM-based classification, we drew attention to the TCGA and GSE58812 cohorts, which comprised 142 and 107 TNBC samples, respectively. First, we calculated the FAM score for each patient and then categorized patients into high- and low-FS subgroups based on the cutoff value of their individual FS. Familiar to the results of METABRIC-TNBC dataset, prognostic analysis also revealed FS-low group was remarkable related to prolonged survival, while FS-high group was characterized by poorer survival (Supplementary Figure 2A, 2B and Supplementary Figure 3A, 3B). In addition, multivariate Cox regression model for TCGA-TNBC cohort supported that FS could serve as an independent prognostic biomarker in TNBC (Supplementary Figure 2C). As for TME immune features, ssGSEA algorithm also identified three distinct immunity phenotypes for TCGA and GSE58812 cohorts (Supplementary Figure 2D and Supplementary Figure 3C). Regarding the immune classification, low-FS subtype also showed greater proportion of high- and medium-immunity (Supplementary Figure 2E and Supplementary Figure 3D). Cibersort and MCPcounter algorithm also revealed that effective immune cells were markedly abundant in low-FS groups (Supplementary Figure 2F, 2G and Supplementary Figure 3E, 3F). ESTIMATE analysis also exhibited higher immune and stromal score in the low-FS group (Supplementary Figure 2H, 2I and Supplementary Figure 3G, 3H). The abundance of immune profiles between different FS subgroups were found a significant difference in TCGA and GSE58812 cohorts (Supplementary Figure 2J and Supplementary Figure 3I). All the above findings demonstrated that the FAM-based classification could be a reliable clinically application for predicting immunotherapy response and prognosis in TNBC.

### Landscape of genomic variation and expression of different FS subgroups in TNBC

**Figure 7:**
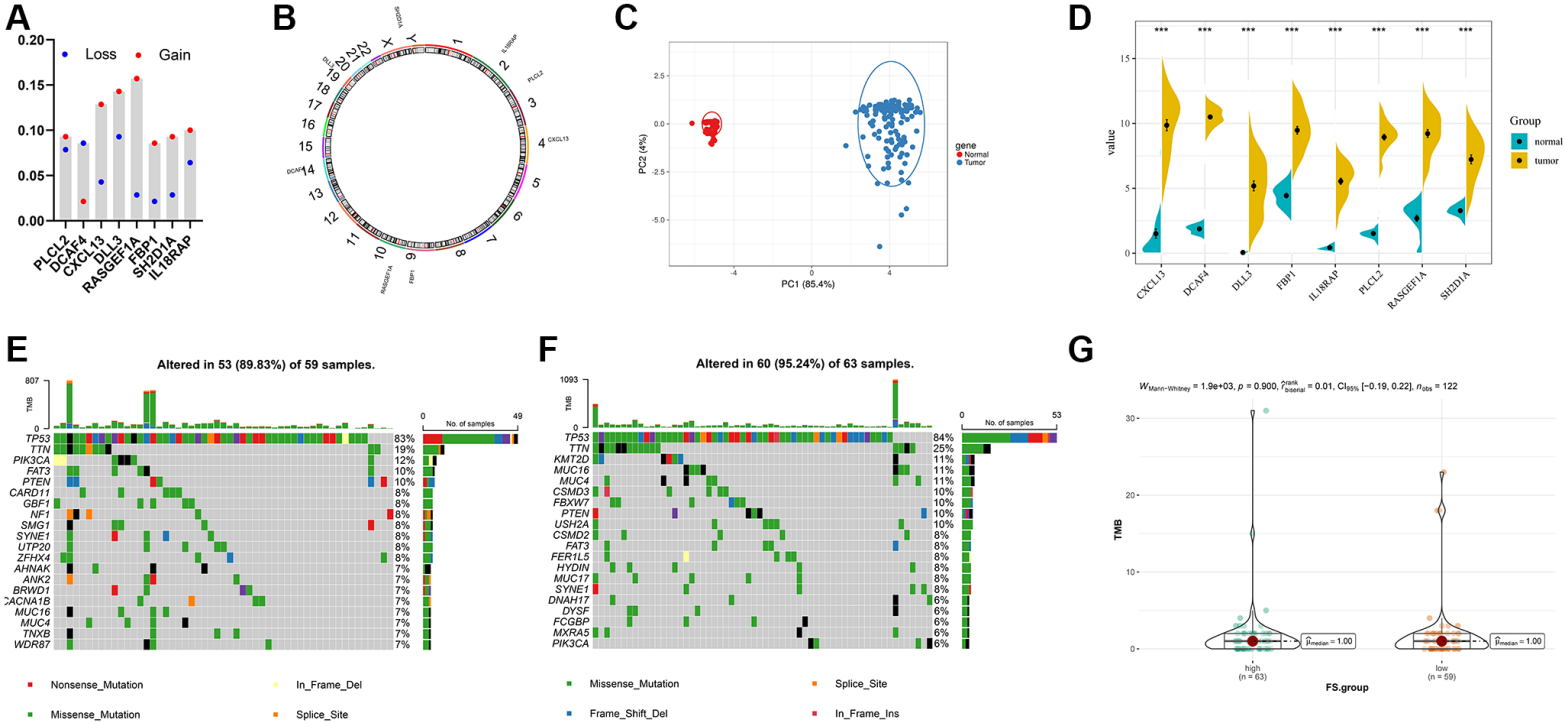
**Landscape of genetic and expression variation of FS in TNBC.** (**A**) The CNV variation frequency of FS gene signatures in TNBC-TNBC cohort. (**B**) The location of CNV alteration of FS gene signatures on 23 chromosomes in TCGA-TNBC cohort. (**C**) Principal component analysis for the expression profiles of 8 FS gene signatures to distinguish tumors from normal samples in TCGA-TNBC cohort. (**D**) The expression of 8 FS gene signatures between normal and tumor tissues. (**E**, **F**) The waterfall plot of tumor somatic mutation of the low- (**E**) and high-FS subgroups (**F**) in TCGA-TNBC cohort. (**G**) The TMB status between the low- and high-FS subgroups in TCGA-TNBC cohort.

First, we recapitulated the frequency of copy number variations (CNV) of 8 selected FS gene signatures in TCGA-TNBC samples (Figure 7A). The locations of CNV alterations of these mutated FAM genes on chromosomes are shown in Figure 7B. Then, we evaluated whether the differential expression of 8 selected FS gene signatures could distinguish TNBC samples from normal samples in TCGA cohort by performing principal component analysis (PCA) (Figure 7C). Furthermore, the mRNA expression levels of these selected FAM gene signature were depicted in Figure 7D, which shown wide diversity between normal and TNBC samples. Next, we further investigated the allocation diversity of somatic mutation and TMB between different FS subgroups in TCGA-TNBC cohort using the R package “maftools”. As shown in Figure 7E, 7F, the top 20 genes of mutation frequency were significant different between the low- and high-FS subtypes. The TMB score between the low- and high-FS subgroups in TCGA-TNBC cohort were further summarized in Figure 7G. The above results indicated that the potentially complex interaction between genomic variation and FS classification in TNBC.

### The role of FS scheme in anti-PD-1/L1 immunotherapy

**Figure 8:**
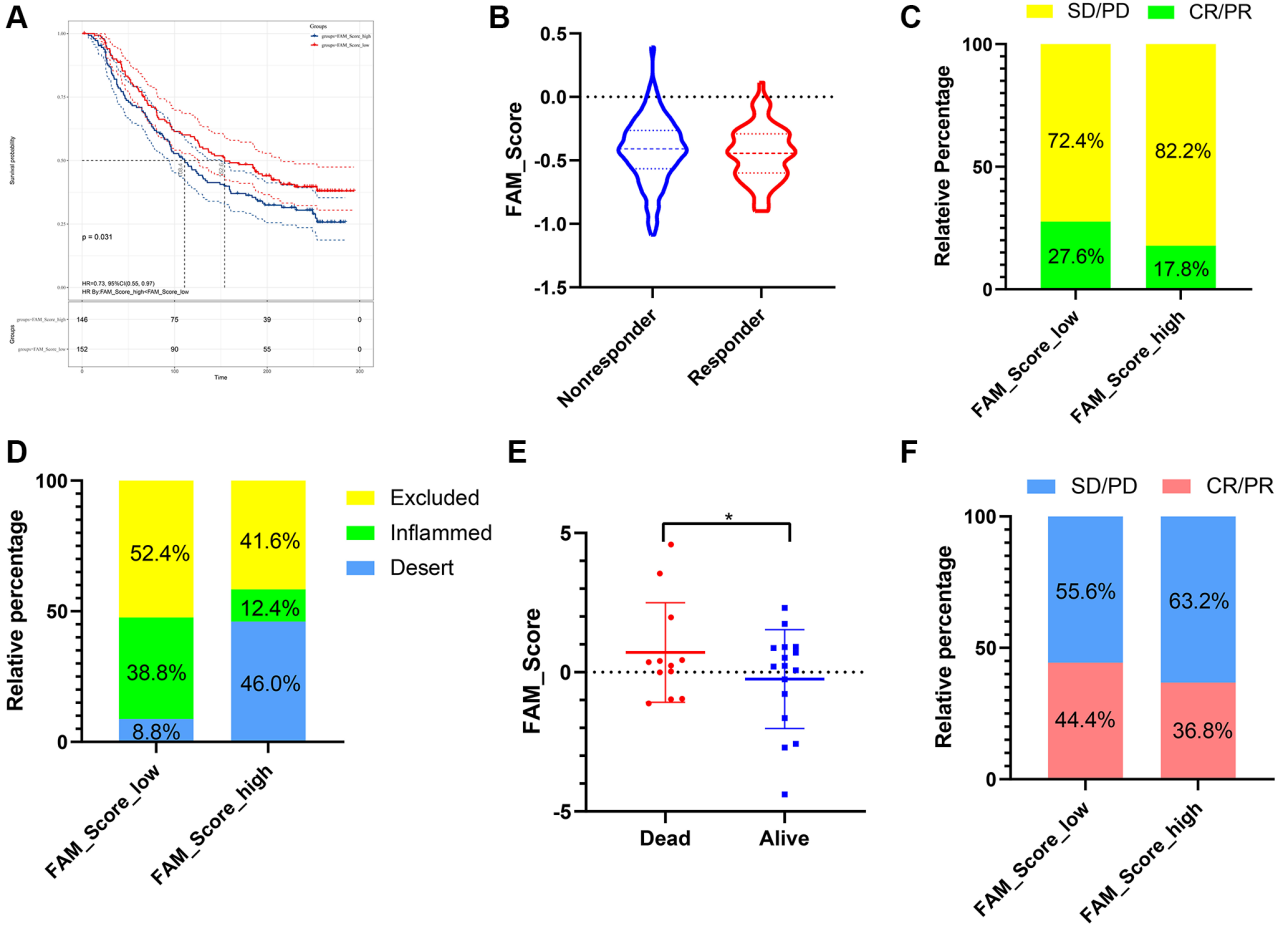
**The role of FS scheme in anti-PD-1/L1 immunotherapy.** (**A**) Survival analyses for low- and high-FS subgroups in the anti-PD-L1 immunotherapy cohort (IMvigor 210). (**B**) The proportion of patients with response/nonresponse to PD-L1 blockade immunotherapy in low and high FS groups (IMvigor 210). (**C**) The proportion of patients with different therapeutic responses to PD-L1 blockade in low and high FS groups (IMvigor 210). (**D**) The proportion of patients with different immunity phenotypes in low and high FS groups (IMvigor 210). (**E**) The proportion of patients with different survival status in low and high FS groups (GSE78220). (**F**) The proportion of patients with different therapeutic response to PD-1 blockade immunotherapy in low and high FS groups (GSE78220). Abbreviations: SD: stable disease; PD: progressive disease; CR: complete response; PR: partial response.

Immunotherapies represented by PD-L1 and PD-1 inhibitors were strongly recommended in antitumor therapy of advanced TNBC patients. Based on two immunotherapy cohorts (IMvigor210 and GSE78220), we next investigated the predictive ability of the established FS system for patients’ response to immune checkpoint blockade. Patients with lower FS exhibited higher clinical benefit rates (Figure 8B, 8C and 8F) and better survival outcomes (Figure 8A, 8E). In addition, patients with higher FS were observed abundant with desert immune phenotype, whereas patients with lower FS were enriched in inflamed immune phenotype (Figure 8D). In summary, the above findings implied that the established FAM based classification could be a practical and promising biomarker for predicting immunotherapy efficiency and survival outcomes in TNBC.

### Identification of novel candidate compounds targeting the selected FAM-related gene signatures

As shown in Supplementary Figure 4, robust positive correlation was found between the expression level of SH2D1A with IC50 of Nelarabine, Dexamethasone Decadron, Fluphenazine and Asparaginase. The IC50 of Fulvestrant and Raloxifene appeared to be positively associated with the expression level of FBP1, similar results were found in the IC50 of Nelarabine and Hydroxyurea with the expression level of PLCL2. A significantly positive correlation was noted between the expression level of IL18RAP and IC50 of Imatinib (all *p* < 0.001). These findings may help to exploring novel treatment strategies for targeting the FAM-related gene signatures in TNBC patients.

### The mRNA levels and prognostic value of selected FAM-related genes in our cohort

**Figure 9:**
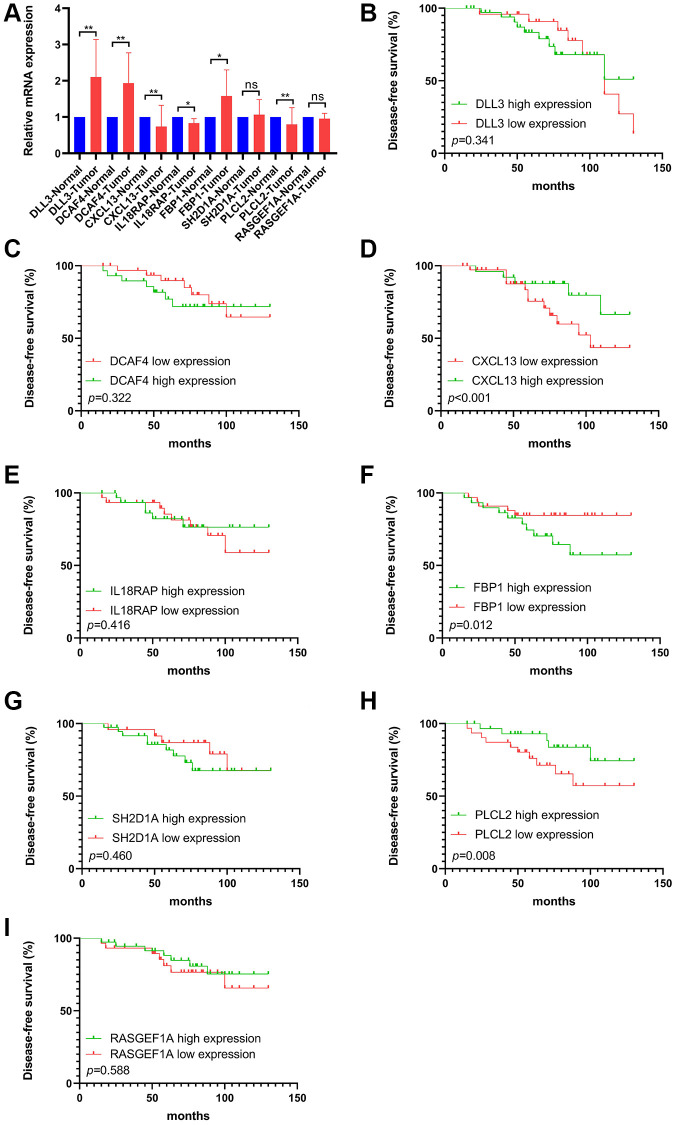
**The mRNA levels and prognostic value of selected FS gene signatures in our cohort.** (**A**) Comparison of mRNA expression levels of selected FS gene signatures in adjacent normal tissues and Tumor tissues by RT-qPCR assay. Kaplan–Meier curve shows the survival diversity between differential expression of DLL3 (**B**), DCAF4 (**C**), CXCL13 (**D**), IL18RAP (**E**), FBP1 (**F**), SH2D1A (**G**), PLCL2 (**H**) and RASGEF1A (**I**) in our cohort. Non-significant (ns) *P* > 0.05, ^*^P < 0.05, ^**^P < 0.01.

The RT qPCR assay exhibited the relative mRNA expression level of selected FAM-related genes (PLCL2, DLL3, DCAF4, CXCL13, RASGEF1A, FBP1, SH2D1A and IL18RAP) in TNBCs and adjuvant normal tissues (Figure 9A). Generally, CXCL13, IL18RAP and PLCL2 mRNA were downregulated, while DLL3, DCAF4 and FBP1 were upregulated in TNBC samples compared with that in the paired ANTs. Furthermore, Kaplan-Meier curve implied that high expression of FBP1 was correlated with worse DFS, whereas low expression of CXCL13 and PLCL2 were associated with worse DFS (Figure 9B–9I).

## DISCUSSION

Studies on classification of tumors based on their FAM relevant profiles are beginning to emerge [34, 35]. Increasing evidence demonstrated that FAM play an indispensable role in the tumorigenesis, immunity regulation as well as chemoresistance in TNBC [36–38]. Thus, elucidating the FAM profiles in shaping immune contexture and heterogeneous of TNBC will providing insights into the interaction of FAM and TME, and steering more efficient immunotherapy strategies for TNBC. To date, the association between FAM and the overall TME infiltration characterizations and heterogeneity of TNBC has not been comprehensively recognized.

In this study, we successfully classified TNBCs into two heterogeneous subtypes defined by their intact FAM features, with distinct survival outcomes, genomic alternations, immune profiles. Further analyses highlighted the FS scheme could serve as an independent prognostic biomarker for predicting survival in TNBC. In accordance with previous studies, we demonstrated that TNBC displayed distinct immune phenotypes among different FAM features [38]. More importantly, the FAM-based classification we constructed could predict the response to anti-PD-1/PD-L1 immunotherapy in two cohorts.

With the aim of identifying the molecular drivers of distinct FS subtypes in TNBC, we observed that significantly mutated genes in different FS groups. The top 20 genes of mutation frequency were significant different between the low- and high-FS subtypes. Moreover, the 8 FS gene signatures could markedly identify tumor tissues from normal breast tissues. We next confirmed the mRNA levels of these FS gene signatures by RT-qPCR assay in our cohort. In accord with the bioinformatics results, the selected FS genes were found differentially expression in TNBC samples. Kaplan–Meier survival analysis showed that differential expression of CXCL13, FBP1 and PLCL2 were remarkably correlated with survival outcomes in TNBCs.

Among the selected gene signatures, DLL3 has been found high expression in breast cancer and was an independent prognostic factor for OS [39]. The expression level of CXCL13 has been found linked to the proinflammatory features of macrophages, could predict the response to the combination of chemotherapy with checkpoint inhibitors for TNBCs [40]. Moreover, previous researches showed that increasing the expression of Aging-associated and CD4 T cell-dependent ectopic CXCL13 were correlated with immune-related adverse events (irAEs) incidence in ICB-treated patients [41]. FBP1, a gluconeogenesis regulatory enzyme, has been found modulate cell proliferation and chemosensitivity by targeting c-myc in breast cancer [42]. Further mechanism analysis showed that MYC-overexpressing TNBC relied on fatty acid oxidation (FAO), inhibition of FAO may as a potential treatment strategy for MYC-overexpressing TNBC. Other studies found that FBP1 deletion disrupted liver metabolic homeostasis and promotes tumorigenesis by inducing the senescence of hepatic stellate cell (HSCs), which established an essential crosstalk between metabolic reprograming and HSC senescence [43]. The interesting findings yielded several novel insights for the molecular drivers in creating subtype-specific FAM reprograming in TNBC.

Our study also has important implications for clinical translations. First, novel therapeutic strategies targeting FAM vulnerabilities are warranted according to subtype-specific features for individual TNBC patients. Second, although immune checkpoint inhibitors have been shown to be successful across multiple tumor types, including TNBC. However, the response to immunotherapy is still low in this tumor type [44, 45], highlighting other underlying mechanisms may influence the immune responsiveness. Mounting evidence have shown metabolites in the tumor microenvironment affecting the fate of immune cells, and therefore, modulate immune responses [46, 47]. On the basis of the FAM features in TNBC, we hypothesized that targeting FAM therapeutic strategies and anti-PD-1/PD-L1 immunotherapy could have a synergistic effect for TNBCs.

Although we set a novel FAM-based classification for predicting immunotherapy efficiency and survival on TNBC, some limitations are needed to declare. First, the FS scheme was identified by bioinformatic analysis using retrospective data; thus, our findings should be validated by prospective studies with TNBC samples. Besides, owing to shortage of an appropriate ICI-based TNBC dataset, the effects of FS scheme on immunotherapy should be further verified to strengthen our conclusion.

In conclusion, this work demonstrated the indispensable role of FAM in shaping tumor microenvironment and heterogeneous for TNBC. Evaluating the FAM features of individual TNBC patient will contribute to enhance our cognition of tumor heterogeneity and TME infiltration features in TNBC, then providing new potential therapeutic targets and steering more efficient immunotherapy strategies.

## Supplementary Materials

Supplementary Figures

Supplementary Tables 1, 2 and 4

Supplementary Table 3
