# Correction for: A novel fatty-acid metabolism-based classification for triple negative breast cancer

**DOI:** 10.18632/aging.204638

**Published:** 2023-03-30

**Authors:** Xia Yang, Wen Tang, Yongtao He, Huimin An, Jin Wang

**Affiliations:** 1 Department of Pathology, Sir Run Run Shaw Hospital of Zhejiang University School of Medicine, Hangzhou, China

**Keywords:** fatty acid metabolism, tumor microenvironment, immunotherapy, triple negative breast cancer

**This article has been corrected:** The authors changed the corresponding author to Jin Wang, who played a crucial role in the revision and supervision of this study, (Email: 3l97039@zju.edu.cn). In addition, they updated the information about the funding of the study by the Zhejiang Provincial Natural Science Foundation (LY2.2H160001).

The corrected **corresponding author, email address** and **funding sections** are shown below.

**Xia Yang^1^, Wen Tang^1^, Yongtao He^1^, Huimin An^1^, Jin Wang^1^**

^1^Department of Pathology, Sir Run Run Shaw Hospital of Zhejiang University School of Medicine, Hangzhou, China

**Correspondence to:** Jin Wang; **email:** 3l97039@zju.edu.cn

