# Supplementary material for: A novel fatty-acid metabolism-based classification for triple negative breast cancer
Source: Aging (Albany NY). 2023 Feb 25;15(4):1177–98. doi: 10.18632/aging.204552 (PMC10008496; doi:10.18632/aging.204552)
Supplement: Supplementary Figures [file aging-15-204552-s001.pdf]

SUPPLEMENTARY FIGURES

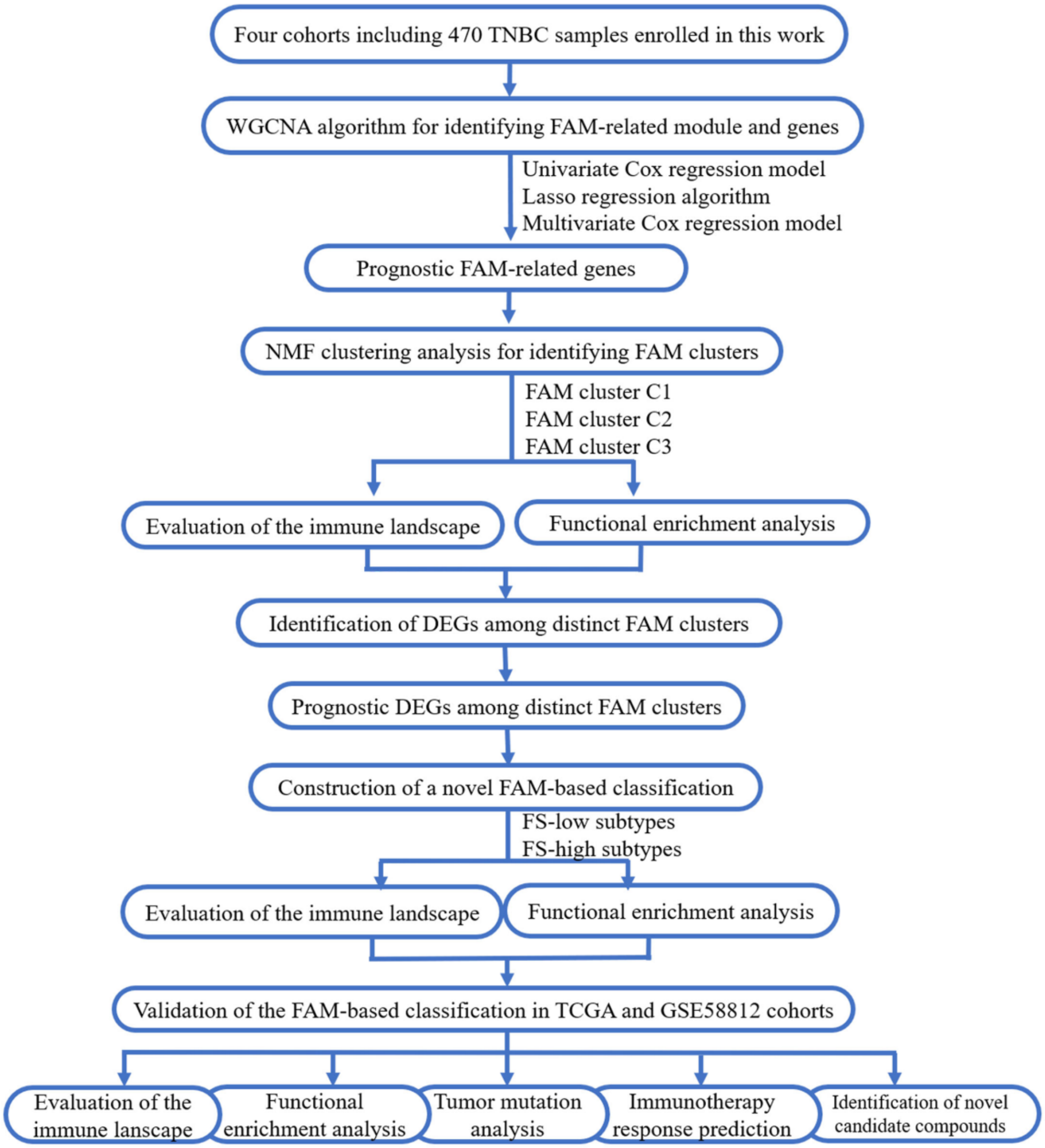

Supplementary Figure 1. The workflow of this study.

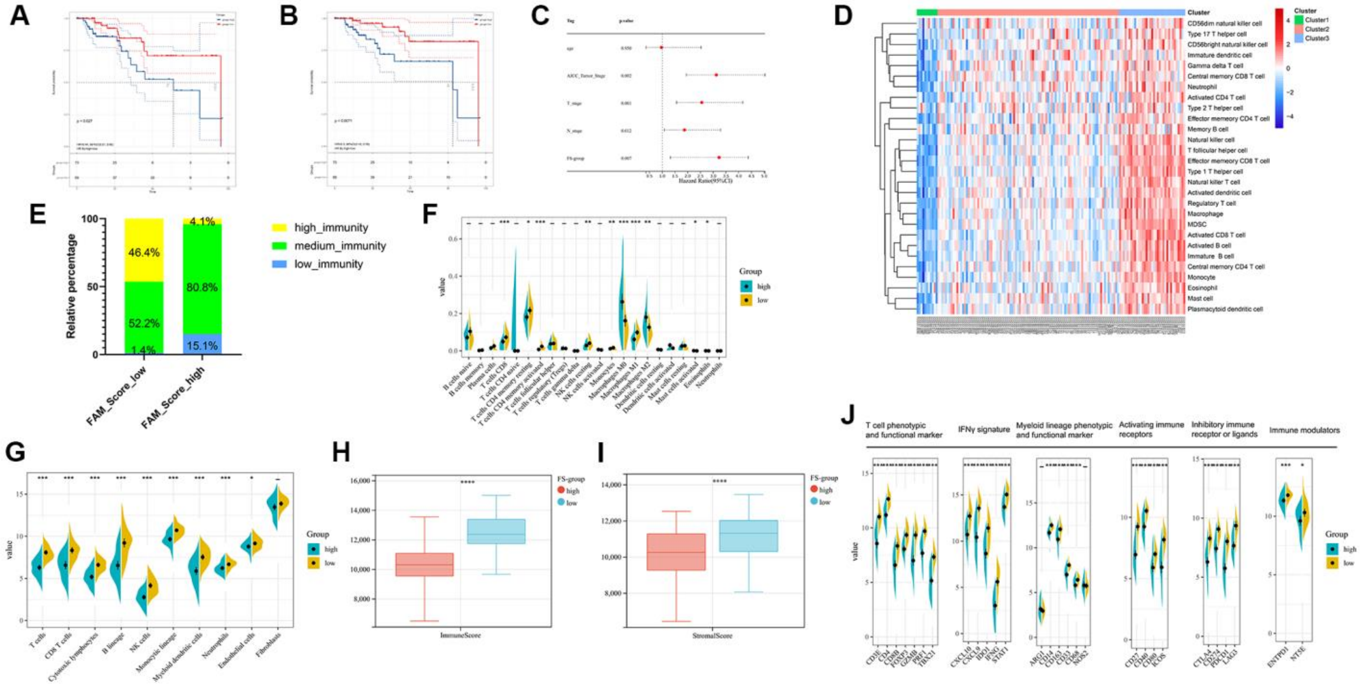

Supplementary Figure 2. (**A**, **B**) Survival analyses for DFS (**A**) and OS (**B**) between low- and high- FS groups in TCGA-TNBC cohort. (**C**) Multivariate Cox regression analysis confirmed that FS could serve as an independent prognostic biomarker for TCGA-TNBC samples. (**D**) ssGSEA showed three distinct immunity phenotypes were identified in TCGA cohort. (**E**) The rate of different immunity phenotypes between the low- and high FS groups. (**F**) Cibersort revealed the abundance of each TME infiltrating cells between the low- and high-FS groups. (**G**) MCPcounter revealed the abundance of each immune infiltrating cell types between the low- and high-FS groups. (**H**, **I**) ESTIMATE analysis exhibited the diversity of the immune (**H**) and stromal score (**I**) between the low- and high-FS groups. (**J**) The expression of immune profiles between the low- and high-FS groups in TCGA cohort.

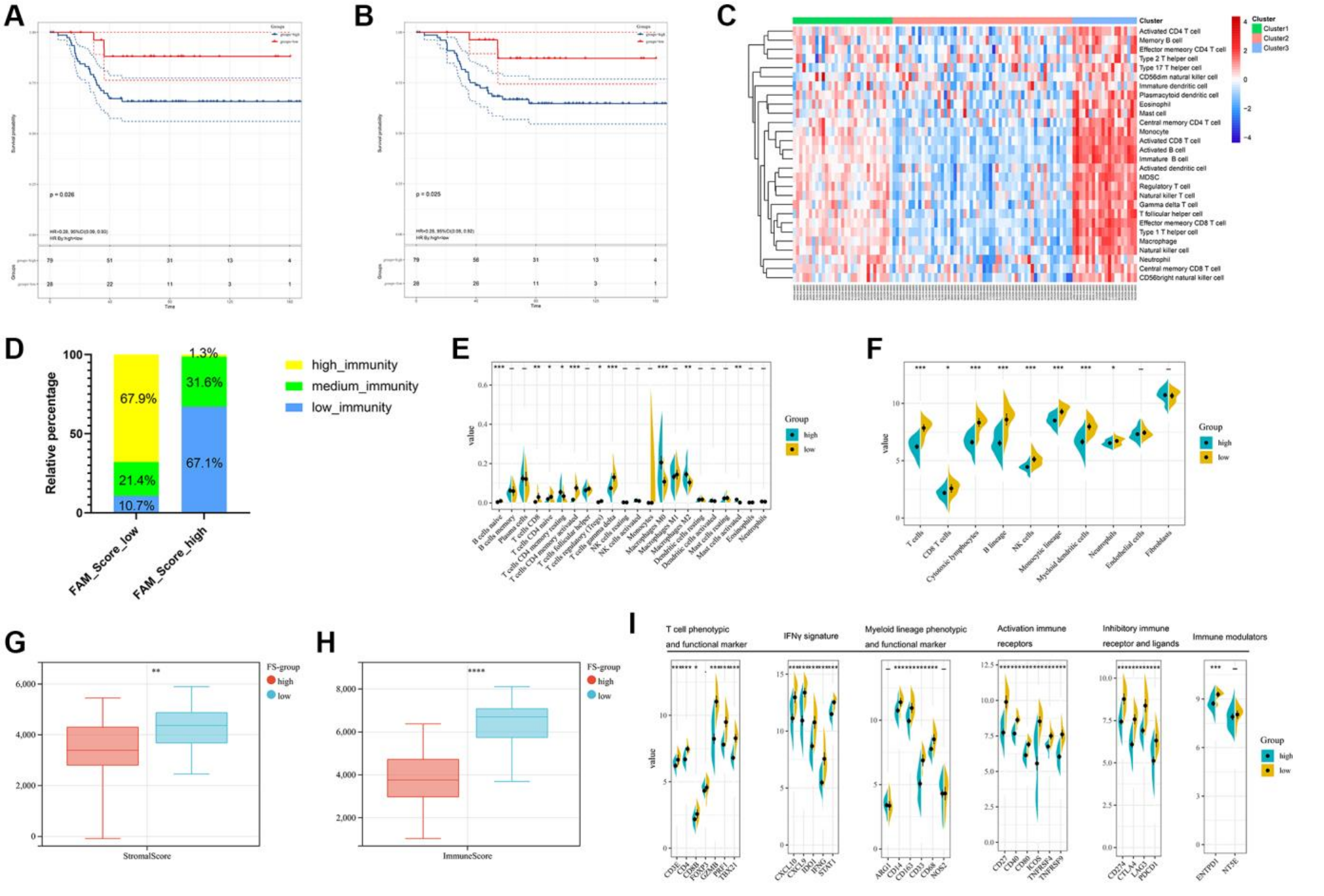

Supplementary Figure 3. (**A**, **B**) Survival analyses for MFS (**A**) and OS (**B**) between low- and high- FS groups in GSE58812 cohort. (**C**) ssGSEA showed three distinct immunity phenotypes were identified in GSE58812 cohort. (**D**) The rate of different immunity phenotypes between the low- and high FS groups. (**E**) Cibersort revealed the abundance of each TME infiltrating cells between the low- and high-FS groups. (**F**) MCPcounter revealed the abundance of each immune infiltrating cell types between the low- and high-FS groups. (**G**, **H**) ESTIMATE analysis exhibited the diversity of the immune (**G**) and stromal score (**H**) between the low- and high-FS groups. (**I**) The expression of immune profiles between the low- and high-FS groups in GSE58812 cohort.

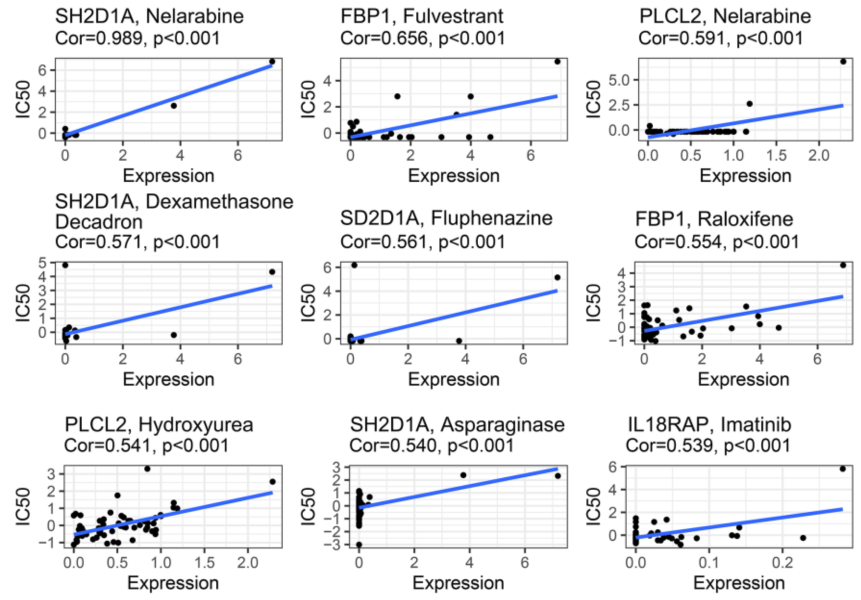

Supplementary Figure 4. Identification of novel candidate compounds targeting the selected FS gene signatures.
