# Supplementary material for: A novel fatty-acid metabolism-based classification for triple negative breast cancer
Source: Aging (Albany NY). 2023 Feb 25;15(4):1177–98. doi: 10.18632/aging.204552 (PMC10008496; doi:10.18632/aging.204552)
Supplement: Supplementary Tables 1, 2 and 4 [file aging-15-204552-s002.pdf]

SUPPLEMENTARY TABLES

Supplementary Table 1. The primers used in this study.

Gene		Sequence (5′ -> 3′)
PLCL2	Forward Primer	ATGACATGATGATTCAGTCCC
	Reverse Primer	GCCTTCTGACAATGTACGA
FBP1	Forward Primer	GATGTTCATCGCACTCTGG
	Reverse Primer	CAGTCTCAGCTTTCCATTGG
DCAF4	Forward Primer	AGTAGAAGACGACATGGGAG
	Reverse Primer	GGAGTCAGACCTGTCTTCAG
CXCL13	Forward Primer	TCCAGTCCAAGGTGTTCTG
	Reverse Primer	CTAGGGATAAAGACTGAGCTCTC
DLL3	Forward Primer	TTCAGAGTCTGCCTGAAGC
	Reverse Primer	TGAAAGAGAAGGTGCCAGG
IL18RAP	Forward Primer	CAGGAGAGCGAATTAAAGGA
	Reverse Primer	CCTTGTAGAATATGTCCAAAGGAG
SH2D1A	Forward Primer	CATTGTAATACCTCTGCAGTATCC
	Reverse Primer	TCTTCTCTTATCCCTGTAGTACC
RASGEF1A	Forward Primer	TGACTTCCAGGATGAGAAGG
	Reverse Primer	GTGCCATTCTCCTCATCAC

Supplementary Table 2. The prognostic values of FAM-related genes.

Univariate-Cox analysis for FAM genes	Lasso analysis for FAM genes	Multivariate-Cox analysis for FAM genes
Hugo_Symbol	Hugo_Symbol	Hugo_Symbol
TBC1D19	TAOK2	DCAF4
MYCN	OPRM1	DEXI
TAOK2	DCAF4	RASGEF1A
OPRM1	LAGE3	OSBP
SESTD1	CREBZF	UTP20
ATP2A2	DEXI	C2CD2L
DCAF4	TRIB2	TYW1
SHCBP1	SLC12A1	SPAG6
SCP2	ADH7	
TPCN1	APOL1	
MMP15	RBKS	
LAGE3	USP30	
MYH6	DMXL2	
XRN1	RASGEF1A	
B4GALT5	OSBP	
CREBZF	ZNF599	
LHPP	UTP20	
CASP6	ZFAND6	

ZBTB10
DEXI
TRIB2
PGAP1
FBXL8
FOXP1
SLC12A1
SENP2
HDLBP
ADH7
TAS1R1
C3orf52
APOL1
FXR1
RBKS
MAP3K1
SNAP23
CTNNB1
SCCPDH
USP30
DMXL2
RASGEF1A
RNF121
DYM
HSPBP1
ZNF524
OSBP
GTF2IRD2
ZNF599
UTP20
ZFAND6
TBCA
TMEM190
DYNLL1
ACOT2
PNMA1
C2CD2L
TYW1
STAR
KRR1
SPAG6
PDCD2

TMEM190
C2CD2L
TYW1
SPAG6
PDCD2
ARRB1
RPGRIP1
riskScore
risk
Gene
Coef

ARRB1
ERI2
DCTN3
SF3B5
RPGRIP1
C12orf45
C17orf100

Supplementary Table 4. Correlation between FS-group and OS in TNBCs.

Tag	*p* value	HR	Low 95% CI	High 95% CI
Age	0.95	0.97	0.37	2.52
AJCC_Tumor_Stage	2.40E-03	3.12	1.94	5.03
T_stage	0.001	2.55	1.56	4.16
N_stage	1.20E-02	1.87	1.08	3.29
FS-group	0.007	3.23	1.32	4.37
